# Family and School Context: Effects on the Mental Health of Brazilian Students

**DOI:** 10.3390/ijerph17176042

**Published:** 2020-08-20

**Authors:** Daise Fernanda Santos Souza Escobar, Thais Ferreira de Jesus, Priscilla Rayanne e Silva Noll, Matias Noll

**Affiliations:** 1 Instituto Federal Goiano, Campus Ceres, Ceres 76300-000, Brazil; daise.ifgoianoceres@gmail.com (D.F.S.S.E.); thaisferreiradejesus@hotmail.com (T.F.d.J.); priscilla.silva@ifgoiano.edu.br (P.R.eS.N.); 2 Universidade de São Paulo, São Paulo 01246-903, Brazil

**Keywords:** adolescents, family context, family influence, mental health, school

## Abstract

Mental health during adolescence can affect an individual’s long-term quality of life. However, the effects of family and school contexts on students’ mental health have been little explored. This study aims to analyze the relationships between family and school life and feelings of loneliness and trouble sleeping owing to worries in adolescents. The data from this cross-sectional study were obtained from Brazil’s National School Health Survey (PeNSE), which obtained its data through questionnaires. This study’s sample consisted of 102,072 ninth-grade students aged between 11 and 19 years, 52,782 (51.7%) of whom were female, enrolled in public and private schools throughout Brazil. The methodology consisted of an analysis using the Poisson regression model. Regarding the family context, mental health issues were associated with hunger, distant relationships with parents, and family violence. Regarding the school context, feelings of loneliness and trouble sleeping were associated with poor peer relationships, insecurity at school, and schools in more violent areas. This study contributes to the elaboration of public policies aimed at bringing awareness to family members and school bodies that indicators of mental health in adolescents are influenced by the quality of bonds established within these environments.

## 1. Introduction

Mental health is related to an individual’s social and physical health and is of global priority [1,2]. The World Health Organization (WHO) defines mental health as the complete physical, social, and mental well-being of an individual [3]. Adolescence, including the ages between 10 and 19 years, is the phase in which changes in mental health usually occur [3,4]. Half of all mental health disorders begin by the age of 14 years, but most cases are not detected or treated at all [5]. Adolescence is associated with constant psychosocial and physical changes that may be accompanied by insecurity and uncertainty. Therefore, adolescence is a period of risk for the development of mental disorders such as excessive irritability, frustration or anger, depression, anxiety, violent behavior, and physical symptoms [5,6]. Globally, the prevalence of mental health disorders in children and adolescents is between 10% and 20%, according to the WHO [5]. The development of mental health problems in adolescence is strongly related with situations and experiences that a child or adolescent goes through in their social circles, primarily those involving family and school [4,7,8].

Good relationships with family members are important for the psychological health of adolescents. Generally, a family is responsible for establishing the social standards of its members [9]. The aspects of the environment where children and adolescents grow up influence their way of dealing with life. These aspects may include inconsistent caregiving, poor living and socioeconomic conditions, family conflict, poor family discipline and management, and the death of a family member [10]. Additionally, family aspects such as pregnant adolescents, adolescent parents, or those in early and/or forced marriages are associated with a greater risk of mental health conditions [5]. The absence or instability of a family can lead to feelings of dissatisfaction, insignificance, loneliness, and other factors, including problematic amount of freedom and exposure to risky behaviors [10,11]. A study conducted in Norway determined that students being able to talk to their parents was a key factor in their quality of life and suggested that good family life supported mental health in adolescents [12]. In England, a study carried out with 203 students aged 11–16 years showed that greater emotional closeness with parents was associated with a lower risk of behavioral and emotional problems [9]. Moreover, a study involving 18,185 North American adolescents determined that good family relationships during adolescence were related to fewer depressive symptoms, while individuals who experienced poor family cohesion demonstrated more depressive symptoms [11].

Because school is a prominent setting for socialization in the lives of adolescents, social interaction within this environment is a significant contributor to an adolescent’s psychological health. Risk factors for mental health in the school environment include academic failure, bullying, failure of schools to provide appropriate environments to support attendance and learning, and inadequate or inappropriate provision of education [10]. Violence takes place primarily in the school or community setting, and exposure to school violence was related to psychological suffering, illness, and damage to mental and physical health in adolescents [10]. An adolescent’s relationships within the school and home environments influence each other. Risky behaviors can develop as unhelpful coping strategies for adolescents with poor mental health and can severely impact their mental well-being [5,8,10,13]. Therefore, it is necessary to pay attention to the quality of an adolescent’s relationships within these settings to reduce negative experiences and improve their well-being [14]. The mental health of adolescents is increasingly recognized as an area of priority for public health policies, because it is essential for the future health of the entire population [5,15]. For future programs to enhance the well-being of this population, it is critically important that researchers further study the connections between adolescent relationships and mental health [4]. Although the relationships between mental health and family and school settings have previously been investigated, few studies have evaluated nationally representative samples, and this topic was largely assessed only in developed countries. Therefore, the present study aimed to analyze the relationships between family and school life and mental health indicators in adolescents using data from a Brazilian populational survey.

We evaluated feelings of loneliness and trouble sleeping due to worries regarding family and school context as outcomes for mental health. The explanatory variables were composed of indicators from family and school context. No medical consultations were carried out to investigate mental health [16], as this study focused on examining the association between two symptoms, “feeling of loneliness” [17] and “trouble sleeping due to worries” [18], which can both indicate poor mental health status [19]. We hypothesized that family and school settings strongly affected the mental health of students; specifically, we theorized that poor relationships were related to a higher incidence of mental health issues. The results of our study may contribute to the development of public health strategies with the objectives of improving the mental health of adolescents, identifying symptoms that may be related to mental health disorders, and monitoring these students, particularly in developing countries like Brazil. 

## 2. Materials and Methods 

This cross-sectional study was conducted using data from Brazil’s 2015 National School Health Survey (PeNSE), which was a sample survey conducted by the Brazilian Institute of Geography and Statistics (IBGE) in partnership with the Ministry of Health (Ministério da Saúde—MS) and the Ministry of Education and Culture (Ministério da Educação e Cultura—MEC) [20]. PeNSE was approved by the National Health Council (Conselho Nacional de Saúde) and the National Commission on Ethics in Research (Comissão Nacional de Ética em Pesquisa—CONEP) under the protocol No. 1,006,467 of 30 March 2015, which regulates and approves human research [20,21].

The PeNSE sample was expanded to encompass the five major regions of Brazil, including the 26 Federative Units of Brazil, the Federal District, and its capitals and cities [21]. Samples from cities and capitals were obtained randomly and with equal probability. Ninth-grade students aged 11–19 years enrolled in public and private schools throughout Brazil participated in this study from April to September 2015 [21]. One class was selected from each school with an independent sample of students for each stratum. The samples were each calculated using a prevalence parameter of 0.5, a maximum error of 0.03%, and a 95% confidence level [21]. The sample consisted of 120,122 students from 4159 classes in 3040 schools. The sample loss, due to absence on the day of collection or invalid questionnaires, was approximately 15%. Thus, the ultimate sample size was 102,072 students.

The PeNSE data collected between April and September 2015 have been evaluated for various health outcomes and risk behaviors in several studies [16,22,23,24,25,26,27,28,29,30]. The survey’s self-administered questionnaires were answered using smartphones, which were distributed by IBGE technicians to the students present on the day of collection. The students received an explanation on how to use the devices. Students in the sampled classes were invited to answer the questionnaires. The students who agreed to participate filled out Informed Consent Forms.

In the present study, indicators of mental health were analyzed with the following questions: “In the last 12 months, how often have you felt alone?” and “In the last 12 months, how often have you been unable to sleep at night because something worried you too much?” For both questions, the answer options were “Never,” “Rarely,” “Sometimes,” “Most of the time,” and “Always.” These questions were evaluated in this population study as indicators of mental health and were not intended to diagnose the mental health of adolescents [20,31]. The possible answers to each question were grouped into dichotomic variables, resulting in “(1) Never or rarely; (2) Sometimes, most of the time, or always” [32]. 

The explanatory variables included in our study were divided into family context (A) and school context (B), as follows:

(A): “How often did you get hungry because you did not have enough food in your house?”; “You usually have lunch or dinner with your parent or guardian”; “How often did your guardians understand your problems?”; “How many days did you skip classes or school without your parents’ approval?”; “In the last 30 days, how often did your parents or guardians go through your things without your approval?”; and “In the last 30 days, how many times were you physically assaulted by an adult in your family?”

(B): “How often have your schoolmates treated you well and/or been helpful to you?”; “In the past 30 days, how often have some of your schoolmates carved you up, intimidated you to the point where you were offended or humiliated?”; “In the past 30 days, how often did your parents or guardians check whether your homework was done?”; “In the past 12 months, how often was the locality where your school is based considered a risk area for violence?”; and “In the past 30 days, how many days did you not go to school because you did not feel safe there?”

The data for mental health and the influence of family and school relationships were analyzed through descriptive statistics using the Wald chi-squared test (bivariate analysis). The independent variables were analyzed using the Poisson regression model with robust variance. The effect measure used was prevalence ratio (PR) with its 95% confidence intervals (CI). The analyses of the variables were adjusted for the sociodemographic variables using the Statistical Package for the Social Sciences (SPSS) version 23.0 (IBM Corp., Armonk, NY, USA). 

## 3. Results

Included in this study were 102,072 total students, of which 49,290 (48.3%) were male and 52,782 (51.7%) were female. Feelings of loneliness (44.7%) and trouble sleeping due to worries (36.2%) were predominant symptoms among residents in the northern region, females, and older students (Table 1).

The associated results are presented in the subsections (Section 3.1) family context and (Section 3.2) school context.

### 3.1. Family Context 

Feelings of loneliness and trouble sleeping due to worries were associated with hunger, having lunch and dinner with parents 4 or fewer days per week, lack of parents’ understanding regarding students’ problems, skipping classes without the parents’ approval, parents going through belongings without the students’ approval, and being assaulted by relatives (Table 2). All family context variables remained associated with feelings of loneliness and trouble sleeping due to worries after adjusted analysis (Table 3).

### 3.2. School Context

The following school context variables were associated with feelings of loneliness and trouble sleeping due to worries: not being treated well and being intimidated by peers, not being assisted by parents in their homework, school located in a violent area, and skipping school due to an unsafe feeling. Moreover, studying in the afternoons and at night was associated with trouble sleeping (Table 4).

The variables of not being treated well by peers, being intimidated or offended by peers, not being assisted by parents in their homework, and skipping school due to an unsafe feeling remained associated with feelings of loneliness and trouble sleeping due to worries after the adjusted analysis. Moreover, school locations in violent areas were associated with feelings of loneliness, whereas studying in the afternoons, at night, and full time were associated with trouble sleeping due to worries (Table 5).

## 4. Discussion

Our study evaluated the relationships between family and school context and feelings of loneliness and trouble sleeping due to worries, which are factors indicative of mental health issues. Our primary results showed that regarding the family context, mental health issues were associated with feelings of hunger, distant relationships with parents, and family violence. Regarding the school context, feelings of loneliness and trouble sleeping were associated with poor peer relationships, insecurity at school, and school locations in areas with a risk of violence. 

Loneliness is associated with weakened mental health, particularly stress, depression, anxiety, suicidal ideation, and low self-esteem [8,33]. Loneliness is a serious issue for adolescents because those who feel lonely are more vulnerable and more likely to engage in risky behaviors [4,32]. It can impair their quality of life and trigger somatic problems that can last throughout their lifespan [10]. Sauter et al. [32] evaluated loneliness and friendlessness among adolescents; the results determined that one in six students reported being lonely most or all the time or having no close friends, designating this high level of perceived loneliness among adolescents as a global public health problem [32]. Furthermore, changes in sleep quality and behavioral problems are related to mental health, as explained by Short et al. [18] and Xiao et al. [34]. Poor sleep quality can cause psychosomatic problems and can indicate mental suffering [35]. 

Family relationships impacted both variables indicative of mental health in our study. Together, variables such as the lack of shared meals, parents’ lack of understanding regarding students’ problems, parents going through things without students’ approval, and lack of attendance and participation in school activities may illustrate a scenario with affective absence and little communication. Although adolescence is a time of seeking autonomy, the emotional support of an adolescent’s family remains indispensable [5,9]. Having good relationships with family members stimulates dialogue and parental support and encourages students to address problems. Moreover, positive family ties promote various social benefits, including the ability to manage relationships [11]. Additionally, positive family associations can even promote better mental health in adulthood [11] and can have a protective effect against depressive symptoms [36].

Unsafe relationships with parents and relatives impair adolescents’ abilities to satisfy their needs for emotion, affection, and security. Adolescents can feel frightened and alone and can develop a negative image of themselves and others, and they may avoid approaching other people because they fear being rejected [10,11]. Parental absence can foster feelings of anguish, frustration, and loneliness [37]. Additionally, adolescents who experience unstructured families, disagreements, breakups and marital violence between parents, and familial violence are at a greater risk of mental disorders [10,36]. Stressful relationships with family and limited emotional support [35] are related to poor sleep quality and difficulty in falling asleep due to worry.

In the family context, those with lower socioeconomic status and those in historically disadvantaged groups are at a higher risk of mental disorders [2,5,10]. Our data demonstrated that feelings of loneliness and trouble sleeping due to worries were associated with hunger owing to not having enough food at home. Poverty, low salary, and lack of access to services predispose an individual to dissatisfaction and lower quality of life, and consequently, to a greater likelihood of developing psychiatric disorders and adopting risky behaviors [10,38]. Aside from the socioeconomic impacts, food insecurity is also related to poor sleep quality, which may justify trouble sleeping due to worries [39]. Food insecurity and economic fragility have also been associated with suicidal thoughts, drug use, and emotional, behavioral, and depression problems [10,32,40].

In our study, feeling lonely and having trouble sleeping due to worries were also associated with assault by an adult in the family two or more times in the last month. The infliction of pain is commonly used as a disciplinary method in countries that are less financially developed. Nevertheless, exposure to family violence is associated with mental health problems [10]. Moreover, once adolescents are physically assaulted, they may believe that violence is a valid method of solving problems. In this way, family violence can result in the reproduction of violence in other spaces [9,37]. Other problems generated by family violence are the loss of trust in caregivers and affective distance. A teenager assaulted at home may not only become violent outside the home but may also be vulnerable to other types of assault [37].

Regarding school context variables, the students in our study who felt lonely and had trouble sleeping due to worries stated that their schoolmates did not treat them well and were intimidating; this made them feel offended and humiliated most of the time or always, to the point where they did not feel safe in the school environment and skipped school because of this. School violence is a global phenomenon that occurs regardless of a country’s economic development [41]. This behavior most often or almost always fits into the concept of bullying [42]. Bullying is more frequent among students who are excluded or disrespected by their peers and do not feel that they belong to the group [43]. 

School victimization, whether by assault or other forms of violence, impacts the health of adolescents, causing traumatic consequences that can last a lifetime. Conversely, schools environments should contribute to the improvement of mental health outcomes [43]. For example, Finland’s education system has implemented a mental health program in schools that has contributed to the reduction of bullying, victimization, and symptoms of anxiety and depression among its students [44]. In Nigeria, mental health services have been associated with the reduction of depressive symptoms and aggressiveness as well as the promotion of social skills [45]. Duru and Balkis showed that insufficient support from school, friends, and parents was strongly related to exposure to violence and declines in adolescents’ mental health [13], corroborating the results of other studies [4,8,10,13,32]. 

Feelings of loneliness and trouble sleeping due to worries have been used as factors for evaluating mental health; although they do not constitute a clinical view, they need to be addressed. Avoiding loneliness in adolescence is vital, considering the harmful effects of feeling alone during this stage of life. The support of parents, guardians, teachers, and friends alleviates the effects of exposure to both familial and school violence, playing a protective role for mental health [4,8,10,13,41]. Loneliness [33] and poor sleep quality [35] can cause serious physical and psychological problems, worsening mental health in the long term. 

Some limitations should be considered when interpreting the results of the present study. First, our findings do not infer causality. Moreover, these datasets are based on self-reports, and the questionnaire was developed specifically for the Brazilian National Survey. We did not measure psychiatric disorders but rather indicators of mental health from issues related to insomnia and loneliness. For future studies, we recommend evaluating a range of mental health indicators and performing longitudinal studies. Moreover, impacts on mental health, not only of adolescents but of their family unit, require further investigation. Our results highlight the necessity of interventions instructing parents, guardians, and teachers to participate more actively in the lives of adolescents.

## 5. Conclusions

The family and school contexts have great influences on the mental health of students. Regarding family context, socioeconomic fragility, and lack of proximity or bad relationships with relatives are associated with feelings of loneliness and trouble sleeping due to worries. In the school setting, bad experiences with peer relationships, not feeling safe at school, and school locations in violent areas are associated with both of the aforementioned indicators of mental health issues. Our results may contribute to the development of mental health prevention strategies. We suggest the adoption of actions to ensure that parents, teachers, and fellow peers are aware of students’ current situations and the consequences of affective distance and violence. Furthermore, we encourage future studies to address other associations, such as the relationship between geographical location and mental health.

## Figures and Tables

**Table 1:** Prevalence of loneliness and trouble sleeping due to worries among ninth grade Brazilian students.

Sociodemographic Variables	Total	Prevalence: “Felt Lonely”		Prevalence: “Trouble Sleeping Due to Worries”	
	%	%	*p*	%	*p*
Region					
South	9.6	44.3	>0.001	35.0	>0.001
Southeast	17.4	43.3		35.2	
Center–West	13.9	44.9		35.2	
North	23.5	45.8		37.7	
Northeast	35.6	44.8		36.9	
City					
Not a capital	49.9	44.0	>0.001	36.2	0.918
Capital	50.1	45.4		36.2	
School					
Public	79.5	44.3	>0.001	36.2	0.860
Private	20.5	46.2		36.3	
Gender					
Male	48.2	32.7	>0.001	26.7	>0.001
Female	51.8	55.9		45.1	
Age					
≤13 years	16.9	43.5	>0.001	32.9	>0.001
14 years	50.6	44.4		35.1	
15 years	20.4	45.2		38.5	
≥16 years	12.0	47.0		41.6	
Mother’s education					
Did not study	7.2	47.6	>0.001	41.8	>0.001
Elementary school *	31.6	45.8		37.3	
High School *	31.6	44.8		35.6	
Higher Education *	29.6	44.3		34.9	

PeNSE: National School Health Survey. * Complete or incomplete.

**Table 2:** Family context and prevalence of loneliness and independent variables among ninth grade Brazilian students.

Family Context Variables	Total %	Felt Alone in the Last 12 Months			Trouble Sleeping Due to Worries		
		Prevalence (%)	PR (95% CI)	*p*	Prevalence (%)	PR (95% CI)	*p*
**Family Life**							
Became hungry because there was not enough food at home (last 30 days)							
Never	77.8	41.1	1	>0.001	33.1	1	>0.001
Rarely	10.7	52.0	1.26 (1.24–1.29)		39.6	1.20 (1.17–1.22)	
Sometimes	8.9	62.0	1.51 (1.48–1.54)		53.4	1.61 (1.58–1.65)	
Most of the time	1.6	67.8	1.64 (1.59–1.71)		57.9	1.74 (1.68–1.82)	
Always	0.9	55.1	1.34 (1.26–1.42)		49.9	1.51 (1.41–1.61)	
Had lunch or dinner with the family							
5 or more days per week	72.7	40.2	1	>0.001	32.7	1	>0.001
1 to 4 days a week	7.2	51.6	1.28 (1.25–1.32)		40.5	1.24 (1.20–1.28)	
Rarely or never	20.1	58.7	1.46 (1.44–1.48)		47.3	1.48 (1.42–1.47)	
Parents or guardians understood your problems (last 30 days)							
Always	27.4	30.4	1	>0.001	28.7	1	>0.001
Most of the time	15.5	35.7	1.17 (1.14–1.21)		29.9	1.04 (1.01–1.07)	
Sometimes	22.5	46.7	1.54 (1.50–1.57)		37.7	1.30 (1.27–1.34)	
Rarely	16.6	55.4	1.82 (1.78–1.86)		40.8	1.42 (1.39–1.46)	
Never	17.9	62.2	2.05 (2.00–2.09)		47.6	1.66 (1.62–1.70)	
Skipped classes or school without parents’ approval (last 30 days)							
None	78.8	43.5	1	>0.001	34.5	1	>0.001
1 to 5 times	19.0	48.5	1.12 (1.10–1.13)		41.8	1.21 (1.19–1.23)	
6 or more times	2.1	57.2	1.32 (1.27–1.37)		51.3	1.49 (1.42–1.55)	
Parents or guardians have gone through your things without approval (last 30 days)							
Never	47.1	39.5	1	>0.001	31.4	1	>0.001
Rarely	21.9	45.2	1.15 (1.13–1.17)		34.4	1.09 (1.07–1.12)	
Sometimes	18.1	52.1	1.32 (1.30–1.34)		43.8	1.39 (1.37–1.42)	
Most of the time	5.3	53.7	1.36 (1.32–1.40)		45.3	1.44 (1.40–1.49)	
Always	7.5	52.3	1.33 (1.29–1.36)		46.7	1.49 (1.45–1.53)	
Physically assaulted by an adult of the family (last 30 days)							
Never	85.8	42.2	1	>0.001	33.6	1	>0.001
1 time	6.5	59.9	1.42 (1.39–1.45)		50.3	1.50 (1.46–1.53)	
2 or more times	7.7	60.6	1.44 (1.41–1.47)		53.3	1.58 (1.54–1.62)	

PeNSE: National School Health Survey; PR: prevalence ratio; CI: confidence interval.

**Table 3:** Family context and adjusted analysis of loneliness and independent variables among ninth grade Brazilian students.

Family Context Variables	Felt Alone in the Last 12 Months		Trouble Sleeping Due to Worries	
	PR Adj (95% CI)	*p*	PR Adj (95% CI)	*p*
Became hungry because there was not enough food at home (last 30 days)				
Never	1	>0.001	1	>0.001
Rarely	1.27 (1.25–1.30)		1.21 (1.17–1.23)	
Sometimes	1.46 (1.43–1.49)		1.55 (1.52–1.59)	
Most of the time	1.53 (1.47–1.59)		1.60 (1.53–1.67)	
Always	1.37 (1.28–1.46)		1.48 (1.39–1.58)	
Had lunch or dinner with the family				
5 or more days per week	1	>0.001	1	>0.001
1 to 4 days a week	1.25 (1.22–1.28)		1.23 (1.19–1.26)	
Rarely or never	1.39 (1.36–1.41)		1.35 (1.32–1.37)	
Parents or guardians understood your problems (last 30 days)				
Always	1	>0.001	1	>0.001
Most of the time	1.20 (1.16–1.23)		1.06 (1.03–1.10)	
Sometimes	1.54 (1.51–1.58)		1.30 (1.27–1.33)	
Rarely	1.79 (1.74–1.83)		1.37 (1.34–1.41)	
Never	2.02 (1.97–2.06)		1.59 (1.55–1.63)	
Skipped classes or school without parents’ approval (last 30 days)				
None	1	>0.001	1	>0.001
1 to 5 times	1.14 (1.12–1.16)		1.22 (1.20–1.25)	
6 or more times	1.34 (1.28–1.39)		1.47 (1.41–1.53)	
Parents or guardians have gone through your things without approval (last 30 days)				
Never	1	>0.001	1	>0.001
Rarely	1.14 (1.12–1.16)		1.10 (1.08–1.13)	
Sometimes	1.31 (1.28–1.33)		1.39 (1.37–1.42)	
Most of the time	1.37 (1.33–1.41)		1.45 (1.41–1.42)	
Always	1.30 (1.26–1.33)		1.46 (1.42–1.49)	
Physical assault by an adult of the family (last 30 days)				
Never	1	>0.001	1	>0.001
1 time	1.36 (1.33–1.39)		1.43 (1.40–1.47)	
2 or more times	1.43 (1.40–1.46)		1.57 (1.53–1.60)	

PeNSE: National School Health Survey; PRadj: prevalence ratio adjusted by sociodemographic variables; CI: confidence interval.

**Table 4:** School context and prevalence of loneliness and independent variables among ninth grade Brazilian students.

School Context Variables	Total	Felt Alone in the Last 12 Months			Trouble Sleeping Due to Worries		
	%	Prevalence (%)	PR (95% CI)	*p*	Prevalence (%)	PR (95% CI)	*p*
School shift							
Morning (7 am to 11 am)	61.6	44.6	1	0.059	35.2	1	>0.001
Afternoon (1 pm to 5 pm)	36.4	44.8	1.01 (0.99–1.02)		37.8	1.05 (1.02–1.09)	
Night (6 pm to 10 pm)	0.1	38.1	0.86 (0.65–1.12)		55.4	2.68 (1.93–3.72)	
Full time (7 am to 5 pm)	1.7	47.8	1.07 (1.02–1.13)		39.5	1.13 (1.00–1.28)	
Between shifts (10 am to 2 pm)	0.2	43.2	0.97 (0.82–1.15)		30.9	0.91 (0.59–1.42)	
Schoolmates treated you well and/or were helpful (last 30 days)							
Always	34.1	37.5	1	>0.001	32.6	1	>0.001
Most of the time	28.6	46.3	1.23 (1.21–1.26)		34.9	1.05 (1.00–1.10)	
Sometimes	20.1	52.3	1.40 (1.37–1.42)		41.6	1.25 (1.19–1.31)	
Rarely	9.4	54.1	1.44 (1.41–1.48)		42.1	1.67 (1.59–1.77)	
Never	7.8	39.8	1.06 (1.03–1.09)		36.1	1.31 (1.23–1.40)	
Intimidated or offended by schoolmates (last 30 days)						
Never	55.8	37.6	1	>0.001	30.8	1	>0.001
Rarely	22.0	47.5	1.26 (1.24–1.28)		36.7	1.38 (1.32–1.44)	
Sometimes	15.5	57.9	1.54 (1.51–1.56)		47.9	1.73 (1.65–1.81)	
Most of the time	3.7	64.4	1.71 (1.67–1.76)		51	2.59 (2.42–2.76)	
Always	3.0	64.2	1.71 (1.66–1.76)		54.7	3.28 (3.08–3.49)	
Parents or guardians checked homework (last 30 days)							
Always	19.8	35.1	1	>0.001	32.6	1	>0.001
Most of the time	12.1	36.8	1.05 (1.02–1.08)		31.6	0.95 (0.89–1.02)	
Sometimes	22.8	42.9	1.22 (1.19–1.25)		37.4	0.93 (0.87–0.98)	
Rarely	19.5	47.9	1.37 (1.33–1.40)		37.4	1.21 (1.15–1.28)	
Never	25.8	55.1	1.57 (1.54–1.60)		41.5	1.52 (1.44–1.59)	
The locality where your school is based was considered a risk area for violence (last 12 months)							
Never	29.5	44.0	1	0.002	35.8	1	0.062
Rarely	18.1	44.2	1.01 (0.98–1.03)		36.3	1.01 (0.99–1.04)	
Sometimes	27.2	45.1	1.03 (1.01–1.04)		36	1.00 (0.98–1.03)	
Most of the time	14.7	45.3	1.03 (1.01–1.05)		37	1.03 (1.01–1.06)	
Throughout the period	10.5	45.9	1.04 (1.02–1.07)		36.8	1.03 (1.01–1.06)	
Skipped school due to not feeling safe (last 30 days)							
None	91.3	43.6	1	>0.001	34.5	1	>0.001
1 to 2 days	5.6	58.8	1.35 (1.32–1.38)		54.6	1.58 (1.54–1.62)	
3 to 4 days	1.7	55.5	1.27 (1.22–1.33)		53	1.54(1.47–1.61)	
5 or more days	1.5	52.7	1.21 (1.15–1.27)		51.1	1.48 (1.40–1.56)	

PeNSE: National School Health Survey; PR: prevalence ratio; CI: confidence interval.

**Table 5:** School context and adjusted analysis of loneliness and independent variables among ninth grade Brazilian students.

School Context Variables	Felt Alone in the Last 12 Months		Trouble Sleeping Due to Worries	
	PR Adj (95% CI)	*p*	PR Adj (95% CI)	*p*
School shift				
Morning (7 am to 11 am)	1	0.780	1	>0.001
Afternoon (1 pm to 5 pm)	1.00 (0.98–1.02)		1.05 (1.04–1.07)	
Night (6 pm 10 pm)	0.96 (0.72–1.27)		1.61 (1.32–1.95)	
Full time (7 am to 7 pm)	1.03 (0.98–1.09)		1.10 (1.04–1.17)	
Between shifts (10 am to 2 pm)	0.99 (0.83–1.18)		0.86 (0.69–1.08)	
Schoolmates treated you well and/or were helpful (last 30 days)				
Always	1	>0.001	1	>0.001
Most of the time	1.26 (1.24–1.29)		1.10 (1.08–1.13)	
Sometimes	1.45 (1.42–1.48)		1.32 (1.29–1.34)	
Rarely	1.49 (1.45–1.53)		1.31 (1.27–1.34)	
Never	1.15 (1.11–1.19)		1.17 (1.13–1.20)	
Intimidated or offended by schoolmates (last 30 days)				
Never	1	>0.001	1	>0.001
Rarely	1.27 (1.25–1.30)		1.22 (1.20–1.25)	
Sometimes	1.54 (1.51–1.57)		1.56 (1.53–1.59)	
Most of the time	1.75 (1.70–1.80)		1.71 (1.65–1.76)	
Always	1.75 (1.67–1.82)		1.82 (1.76–1.88)	
Parents or guardians checked homework (last 30 days)				
Always	1	>0.001	1	>0.001
Most of the time	1.09 (1.06–1.13)		1.01 (0.98–1.04)	
Sometimes	1.25 (1.21–1.28)		1.09 (1.06–1.12)	
Rarely	1.36 (1.32–1.39)		1.14 (1.11–1.17)	
Never	1.51 (1.47–1.55)		1.22 (1.19–1.25)	
The locality where your school is based was considered a risk area for violence (last 12 months)				
Never	1	>0.001	1	0.083
Rarely	1.00 (0.98–1.02)		1.02 (0.99–1.04)	
Sometimes	1.02 (1.00–1.04)		1.01 (0.99–1.05)	
Most of the time	1.02 (1.01–1.05)		1.04 (1.01–1.06)	
Throughout the period	1.04 (1.01–1.07)		1.03 (1.00–1.06)	
Skipped school due to not feeling safe (last 30 days)				
None	1	>0.001	1	>0.001
1 to 2 days	1.32 (1.28–1.35)		1.53 (1.49–1.56)	
3 to 4 days	1.32 (1.26–1.39)		1.58 (1.51–1.65)	
5 or more days	1.30 (1.23–1.37)		1.54 (1.46–1.62)	

PeNSE: National School Health Survey; PR adj: prevalence ratio adjusted by sociodemographic variables; CI: confidence interval.
